# Could Pomegranate Juice Help in the Control of Inflammatory Diseases?

**DOI:** 10.3390/nu9090958

**Published:** 2017-08-30

**Authors:** Francesca Danesi, Lynnette R. Ferguson

**Affiliations:** 1 Department of Agri-Food Science and Technology (DISTAL), University of Bologna, Piazza Goidanich 60, 47521 Cesena, Italy; 2 Discipline of Nutrition and Dietetics, Faculty of Medical and Health Sciences, The University of Auckland, Private Bag 92019, Auckland 1142, New Zealand; l.ferguson@auckland.ac.nz

**Keywords:** pomegranate, *Punica granatum*, pomegranate juice, ellagitannins, inflammation, inflammatory diseases, anti-inflammatory properties

## Abstract

Fruits rich in polyphenols, such as pomegranates, have been shown to have health benefits relating to their antioxidant and anti-inflammatory properties. Using data obtained from PubMed and Scopus, this article provides a brief overview of the therapeutic effects of pomegranate on chronic inflammatory diseases (CID) such as inflammatory bowel disease (IBD), rheumatoid arthritis (RA), metabolic and cardiovascular disorders, and other inflammatory-associated conditions, with an emphasis on fruit-derived juices. Most studies regarding the effects of pomegranate juice have focused on its ability to treat prostate cancer, diabetes, and atherosclerosis. However, pomegranate juice has shown therapeutic potential for many other illnesses. For instance, a small number of human clinical trials have highlighted the positive effects of pomegranate juice and extract consumption on cardiovascular health. The beneficial effects of pomegranate components have also been observed in animal models for respiratory diseases, RA, neurodegenerative disease, and hyperlipidaemia. Furthermore, there exists strong evidence from rodent models suggesting that pomegranate juice can be used to effectively treat IBD, and as an anti-inflammatory agent to treat CID. The effects of pomegranate intake should be further investigated by conducting larger and more well-defined human trials.

## 1. Introduction

Inflammation is a complex biological response to tissue injury and infection. Chronic inflammation has been shown to be involved in the onset and development of a range of disorders. Chronic inflammatory disease (CID) is a general term used for conditions where persistent inflammation plays a central role in disease pathology [1]. Examples of CID include rheumatoid arthritis (RA), inflammatory bowel disease (IBD), chronic obstructive pulmonary disease (COPD), asthma, and psoriasis. Patients with CID present with heavy infiltration of inflammatory cells at the site of disease—e.g., joints, intestinal mucosa, lungs, and skin—and show elevated levels of inflammatory mediators [2]. Dysfunctional inflammatory responses have also been implicated as contributors to other chronic diseases such as atherosclerosis, type 2 diabetes, obesity, insulin resistance, and certain neurodegenerative diseases (e.g., Alzheimer’s disease) [3].

**Figure 1:**
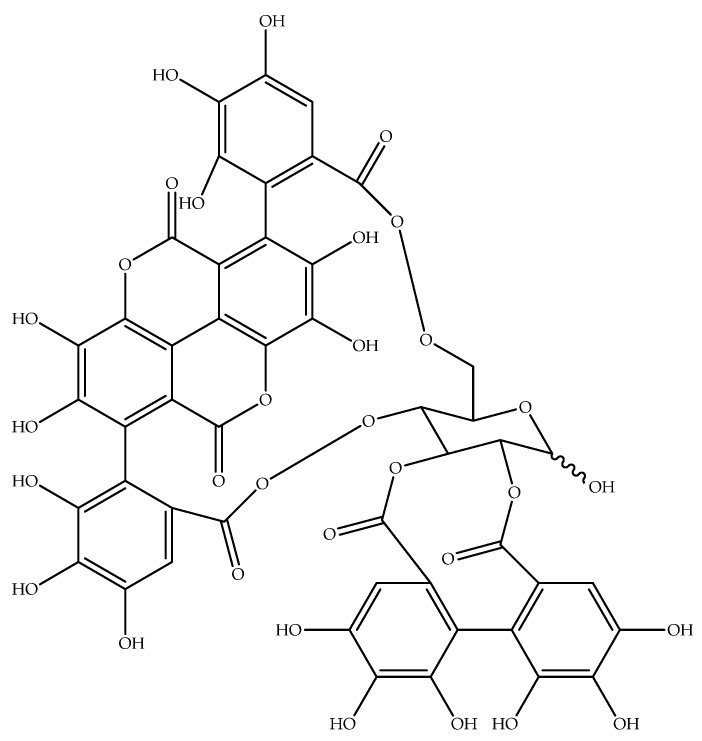
The structure of punicalagin, the main polyphenolic compound present in pomegranate.

**Figure 2:**
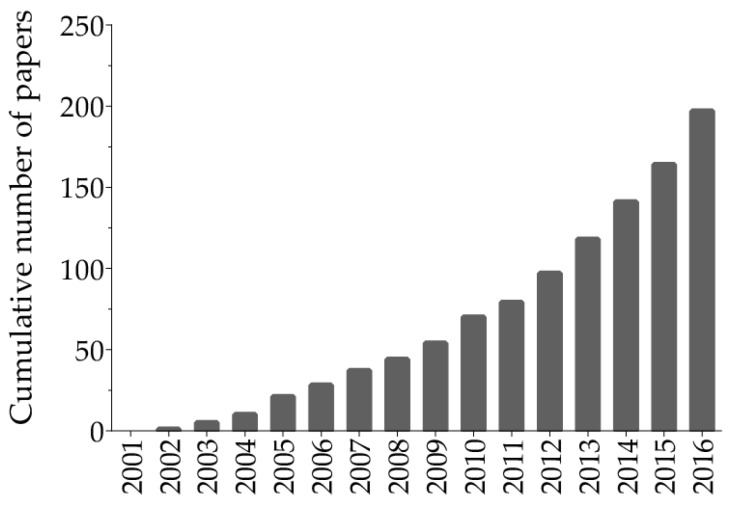
The popularity of health benefits of ellagitannins over time as shown by the cumulative number of papers from 2001 to 2016 obtained by a basic search on the PubMed database using “ellagitannins” and “health” as terms search.

Ellagitannins (ET) and ellagic acid (EA) are polyphenols present in some fruits, nuts, and seeds—such as pomegranates, black raspberries, raspberries, strawberries, walnuts, and almonds [4]. It has been shown that ET-rich fruits have anti-oxidant, anti-inflammatory, anti-neoplastic, and chemo-preventive properties [5]. Pomegranate (*Punica granatum* L.) is a rich source of ET punicalagin (Figure 1), which aroused considerable interest in pomegranate fruit as a novel therapeutic within the last several years (Figure 2).

Although it is widely accepted that pomegranate intake can provide significant health benefits, the results of human clinical trials using pomegranate juice as a therapeutic agent have been inconsistent. This may, in part, be due to variability in the composition of the administered pomegranate products. Several studies have suggested that pomegranate intake has positive effects on blood pressure [6,7] and cardiovascular risk in diabetic [8,9], obese [10,11], hypertensive and ischemic patients [12,13]. Conversely, a meta-analysis found that pomegranate had no effect on lipid profiles [14]. In addition, a meta-analysis of data from five prospective trials did not find a significant effect of pomegranate juice on plasma C-reactive protein (CRP) levels [15].

No clear consensus has yet emerged on the putative anti-inflammatory effects of pomegranate intake on CID. Therefore, we conducted a systematic review to provide an overview of the evidence of the potential benefits of pomegranate products—with an emphasis on fruit-derived juices—on this occurrence.

## 2. Search Strategy

We performed an extensive search using the PubMed and Scopus databases in April 2017. The following keywords and Medical Subject Headings (MeSH) terms were combined: “pomegranate” or “Punica granatum”, “inflam*”, and “disease*”. We did not use language restrictions, and reviews were excluded. The search strategies were as follows: Medline search strategy (pomegranate*) OR (Punica granatum) AND inflam* AND disease* NOT review*; Scopus search strategy (TITLE-ABS-KEY (pomegranate*) OR TITLE-ABS-KEY (Punica granatum) AND TITLE-ABS-KEY (inflam*) AND TITLE-ABS-KEY (disease*)) AND (LIMIT-TO (DOCTYPE, “ar”)).

## 3. Results and Discussion

**Figure 3:**
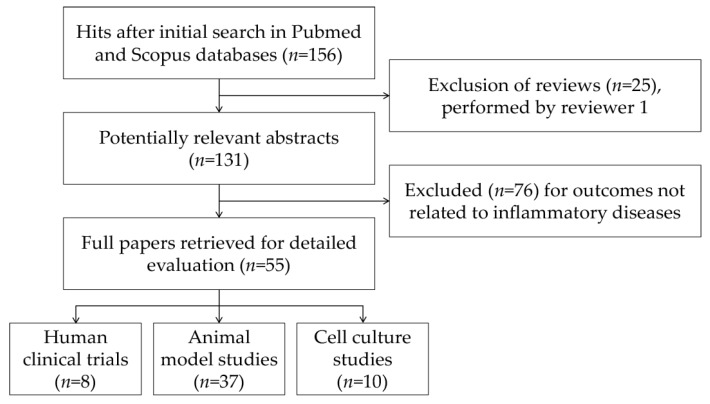
Flow chart of papers included in the review.

The literature selection process was conducted following PRISMA (Preferred Reporting Items for Systematic Reviews and Meta-Analyses) recommendations [16]. The initial search yielded 156 hits after the exclusion of duplicates. During the screening process (reviewing of titles and abstracts), 25 records were excluded. After full-text analysis, another 76 papers were excluded. Altogether, 55 papers were selected for detailed evaluation. Both reviewers independently selected the evaluated articles. Figure 3 shows the flow chart of the selection procedure of the papers.

Human clinical trials are relatively few (Table 1) and were mainly focused on the effects of pomegranate consumption on cardiovascular health.

### 3.1. Findings Related to Pomegranate Products Consumption and CID in Humans

Table 2 summarises the data published on human trials related to pomegranate intake. Unfortunately, there exists a limited number of human trials available concerning the effects of pomegranate on the outcomes of patients with CID. Within these studies, we found the tested dietary products and experimental designs to be highly variable. Only one trial has been conducted using pomegranate seed oil [32], whereas most other studies have been carried out using either pomegranate juice [33,34,35,36,37] or the commercially available whole fruit phenolic extract POMx^TM^ [37,38,64], which are both safe and well-tolerated by CID patients. The doses of pomegranate juice or extract used also varied from study to study as groups used a range of 100 to 500 mL [35] of juice and 0.5 to 1 g of POMx^TM^ [37,38]. Additionally, the study designs used differ for each trial—some used pre- and post-test schemes [33,34] or randomised placebo-controlled tests [32,35,36,38,64], and one trial even lacked an appropriate placebo comparator [37]. Finally, the duration of each study is not consistent—some performed acute (1 day) [33] or short-term (1 week) [35] trials and others assessed the long-term (6–12 months) effects of pomegranate intake [36,38]. While these studies focused primarily on patients affected by metabolic and cardiovascular disorders [32,33,34,35,36,37,38] and RA [64], there exist three clinical trials that are currently in progress exploring the therapeutic potential of pomegranate juice on IBD, memory impairment, ageing and skin inflammation (Table 3).

Even though the use of differing approaches renders it difficult to draw general conclusions, there is a generally positive effect of pomegranate consumption in patients with chronic inflammatory disorders. Pomegranate juice appears to have promising hypotensive properties in patients with hypertension [33] or metabolic syndrome [35,71], and in patients undergoing dialysis [38]. It also resulted in a slight amelioration of lipid profiles in patients with cardiovascular disease (CVD), as pomegranate intake elevated endogenous levels of high-density lipoprotein (HDL)-cholesterol [34] and reduced triglyceride (TG) levels [32]. However, several studies have been unable to confirm pomegranate’s TG- and cholesterol-lowering effect [34,35,37,38]. With regards to risk factors for CVD, the consumption of 150 mL of pomegranate juice—restricted to one serving per day—did not influence the level of circulating soluble adhesion molecules or markers of atherosclerosis and subclinical coronary heart disease (CHD) in hypertensive individuals [33]. Evidence for the beneficial effects of pomegranate on CVD was obtained using long-term consumption (100 mL daily for one year) [36] or with a greater intake of juice (500 mL daily) [35].

Markers of systemic inflammation have not been consistently evaluated in many of these studies, a factor which has an impact on the conclusions drawn from each trial. It has been shown that circulating pro-inflammatory cytokines in patients affected by cardiovascular disorders are slightly reduced by the consumption of pomegranate juice and extract. Notably, a decrease in the level of interleukin 6 (IL-6)—a well-known pro-inflammatory cytokine—has been demonstrated in patients with type 2 diabetes [34] and in patients undergoing haemodialysis [36]. Conversely, pomegranate intake has no effect on the plasma levels of CRP or tumour necrosis factor α (TNF-α), except in patients with metabolic syndrome [35] or those undergoing dialysis [36]. It is generally accepted that the anti-inflammatory effects of pomegranate intake are mediated by its anti-oxidant properties. In agreement, pomegranate intake results in an improvement in plasma antioxidant capacity, as it has been shown to decrease the prevalence of oxidatively damaged molecules and increase anti-oxidant-dependent immune responses in patients affected by CID [34,64].

In conclusion, evidence from human trials indicates that pomegranate, when administered as a juice in high doses or for an extended period, can reduce oxidative stress and systemic inflammation. However, there exist limitations to many of these studies as these trials were limited in sample size, used short durations of supplementation, and/or lacked necessary controls.

### 3.2. Evidence of Anti-Inflammatory Effects of Pomegranate or Pomegranate-Derived Products in Different Animal Models of CID

Animal models have been used to investigate the pathology of a wide range of chronic inflammatory diseases—COPD, IBD, metabolic and cardiovascular disorders, neurodegenerative diseases, RA, cancer—in which inflammation is induced experimentally by genetic manipulation or pharmacologically (diet, drugs, or exogenous toxicants) (Table 4). These studies examined the effects of different pomegranate-related products, including whole fruit juice [19,21,26,41,42,43,48,50,60] or extract [22,24,26,40,43,44,52,55,56,59,67,68,69], extracts obtained using various parts of the fruit (e.g., peel [17,45,47,58], seeds [20,43,46,50,54], flowers [27,45], leaves [18]), concentrated extracts (POMx^TM^ [65], Pomanox^®^ [51]) and bioactive molecules found in pomegranates (punicalagin [53], EA [23,24,25,49,57], punicic acid [20]) and their derived metabolites (urolithins [22]). The administered doses of these extracts varied in range between 10 to 80 mg/kg [24,25,57]. Based on the previous findings of Kaulmann and Bohn (2016) [73], the doses administered in these in vivo studies probably resulted in supra-physiological serum concentrations of pomegranate metabolites obtained only through supplements in humans.

These studies found anti-inflammatory effects of pomegranate and its biologically active compounds. The inflammation-related endpoints measured varied between local—e.g., macroscopic and histological examinations—to systemic evaluations—scoring systems, and plasma cytokines and CRP levels. Inflammation can also be induced by oxidative stress and, accordingly, oxidative markers were often evaluated. Among them, the most commonly measured endpoints included the formation of malondialdehyde (MDA) [22,26,27,53,54,58,68], antioxidant capacity (measured as FRAP or ferric reducing ability of plasma) [22], and the activities of glutathione peroxidase (GPx) [48], superoxide dismutase (SOD) [26,42,48,52,58,68], catalase (CAT) [48,68], and myeloperoxidase (MPO) [20,24,25,26,27]. Typically, the reduction of local inflammation and oxidative stress has also been reported to have more systemic effects.

Administration of pomegranate-derived products has been shown to reduce local inflammation in the bronchoalveolar tissue of COPD model mice [17,18,19] and in the joints of RA model mice [65]. There also exists a strong base of evidence suggesting that pomegranate extract exerts anti-inflammatory effects that may alleviate the symptoms of IBD [20,21,22,23,24,25,26,27], as colon tissue damage [20,22,23,24,25,26,27], antioxidant status [20,22,24,25,26,27], and inflammation [21,22,23,24,25,26] were all ameliorated by pomegranate fruit supplementation in rodent models of IBD. The mechanisms involved appear to be related to the inhibition of NF-κB [23,24,25,26], c-Jun N-terminal kinase (JNK), extracellular signal-regulated kinase (ERK), and signal transducer and activator of transcription 3 (STAT3) phosphorylation [23,24,25] in colon tissue.

Several groups have studied the effects of pomegranate on the prevention and amelioration of atherosclerosis and other CVD symptoms. de Nigris et al. (2007) [43] reported that supplementation with pomegranate juice or pomegranate fruit extract decreased the expression of vascular inflammation markers and transforming growth factor β-1 (TGFβ-1), and, likewise, increased endothelial NO synthase (eNOS) levels in a rat model of metabolic syndrome. Additionally, Labsi et al. (2016) [74] showed that intraperitoneal treatment with pomegranate peel extract for two months after the induction of echinococcosis significantly reduced the nitric oxide (NO) and TNF-α levels in Swiss albino mice. Pomegranate juice supplementation also ameliorated cardiac hypertrophy, and reduced oxidative stress and expression levels of interleukin 1β (IL-1β) and several fibrotic markers in the aorta of rats exposed to cigarette smoke [39]. Similarly, pomegranate juice supplementation slowed the development of non-alcoholic fatty liver disease (NAFLD) and non-alcoholic steatohepatitis (NASH) in a diet-induced obesity rat model [48]. Other authors illustrated the hypoglycaemic activity of pomegranate flowers [45], seeds [46], peel [45], and juice [41,48] in addition to its bioactive compound EA [49] using various rodent models of diabetes. Here, pomegranate-derived products appeared to regulate the activation of peroxisome proliferator-activated receptor γ (PPAR-γ) [46], a known regulator of fatty acid storage and glucose metabolism.

Several studies assessed the efficacy of pomegranate fruit as an antiproliferative agent in animal models of prostate hyperplasia and carcinoma. Malik and Mukhtar (2005) [69] demonstrated that, in vivo, oral administration of pomegranate fruit extract results in tumour growth inhibition accompanied by a reduction in serum prostate-specific antigen (PSA) levels. The decreasing of serum PSA levels have also been confirmed in two studies in humans [75,76]. In addition, dietary supplementation of 4% pomegranate extract with a standard chow diet inhibited neuro-inflammation in a transgenic mouse model of Alzheimer’s disease (AD). A delay in the formation of senile plaques and the loss of synaptic proteins was also observed [55,56]. Conversely, treatment with pomegranate juice did not protect neuronal degeneration in a separate study that used a rat model of AD, but instead exacerbated neuronal cell death and inflammation [60].

In conclusion, these studies using animal models of inflammation-related disease offer a crucial step in answering phenomenological questions related to the pathology of CID, but cannot be fully applied clinically due to the relatively high doses of pomegranate products used.

### 3.3. In Vitro Anti-Inflammatory Activity of Pomegranate Extracts or Pomegranate-Derived Bioactive Compounds

The anti-inflammatory effects observed in vivo have generally been confirmed in vitro. However, experimental conditions applied to cell cultures also tend to vary considerably with regards to the concentration of pomegranate extracts and time-points used (Table 5). In vitro, several pomegranate products have been tested including whole juice [61,63,70], extracts from the husk [29], seeds [62], or pulp [61,77], bioactive compounds present in pomegranate juice (ET [29,53,77] and EA [30]), or POMx^TM^ extract [31]. Of all the groups working with these compounds, only Giménez-Bastida et al. (2012) [28] decided to assess the effects in intestinal cells of EA, which is present at high concentrations in the human colon. It is well known that pomegranate ETs are first hydrolysed to EA followed by transformation into the metabolite—urolithin—in the gut [5]. The importance of identifying bioactive metabolites using cell-based experimentation has been recently elaborated upon by Aragonès et al. (2017) [78].

Throughout our literature review, we found that the concentrations of the compounds applied to cells (mainly ET and EA) ranged from 1 to 100 μM; concentrations like 100 μM are considered high but physiologically attainable in the gut. The duration of supplementation was also variable, as groups used short-term (1–2 h) and long-term exposure to ET, EA, and pomegranate extracts (24–48 h), and were tested prior to exposure or in combination with pro-inflammatory stimuli, such as IL-1β [28,29,63], TNF-α [28,66], interferon γ (IFN-γ) [29], lipopolysaccharide (LPS) [29,53,62], phorbol 12-myristate 13-acetate (PMA) [31,79], or glucose deprivation [61]. In a few cases, pomegranate extracts or bioactive molecules were tested individually [30,70].

As mentioned above, in vivo studies have defined a clear role for NF-κB in the modulation of inflammation by pomegranate extracts, a finding that appears to be confirmed in vitro. Pomegranate juice [63], POMx^TM^ extract [31], and their bioactive compounds—punicalagin [53] or delphinidin [66]—all suppressed NF-κB activation in various types of cells. It was found that ET reduced the expression of NF-κB target genes, including IL-6 and interleukin 8 (IL-8), upon exposure to pro-inflammatory stimuli in intestinal cells [29], while EA [30] and POMx^TM^ [31] reduced NF-κB activation in various subsets of immune cells, and anthocyanin delphinidin reduced inflammation in rheumatoid arthritis cells [66]. Taken together, these results suggest that ET and other bioactive compounds present in pomegranate juice show anti-inflammatory effects in vitro, and that the mechanisms involved appear to be related to inactivation of NF-κB signalling.

## 4. Conclusions and Future Directions

Despite abundant literature on the putative effects of pomegranate fruit or extract on CID and other inflammation-related diseases, a definitive relationship between the consumption of pomegranate products and its beneficial properties has not yet been established. It is likely that the effects are due to the ingestion of pomegranate’s bioactive polyphenolic molecules. To date, most scientific research on the promising health benefits of pomegranate have been carried out in animal or cell culture models. Clinical trials are currently being conducted to examine a wide range of the potential health effects of pomegranate. However, these trials are few. The most promising properties of pomegranate thus far are related to its effects on diabetes, metabolic syndrome, and cardiovascular diseases. Further studies are required to determine the specific effects of ET-containing foods [80], and to explain the health benefits of pomegranate on CID.

## Figures and Tables

**Table 1:** Published research studies on the potential beneficial effects on Chronic Inflammatory Disease (CID) of pomegranate products.

CID	Human Clinical Trials (No. of Subjects)	Animal Model Studies	Cell Culture Studies
Asthma and COPD	-	3 [17,18,19]	-
IBD	-	8 [20,21,22,23,24,25,26,27]	2 [28,29]
Immune system	-	-	2 [30,31]
Metabolic and cardiovascular disorders ^§^	7 (51 [32], 13 [33], 31 [34], 30 [35], 101 [36], 24 [37], 27 [38])	14 [39,40,41,42,43,44,45,46,47,48,49,50,51,52]	-
Neurodegenerative diseases	-	8 [53,54,55,56,57,58,59,60]	4 [53,61,62,63]
Psoriasis	-	-	-
RA	1 (55 [64])	1 [65]	1 [66]
Other disorders ^§§^	-	3 [67,68,69]	1 [70]

COPD: chronic obstructive pulmonary disease; IBD: inflammatory bowel disease; RA: rheumatoid arthritis. ^§^ atherosclerosis, type 2 diabetes, obesity, metabolic syndrome, insulin resistance, hyperlipidaemia. ^§§^ cell proliferation, hyperplasia, metaplasia, cancer.

**Table 2:** Summary of findings related to pomegranate products consumption and CID in humans.

Study Design	Population	Subjects (Gender, No., Age)	Intervention	Control/Comparator	Duration	Outcomes	Reference
Double-blind, placebo-controlled, randomised	Dyslipidaemic patients	F & M: 51, 42–64 years	Pomegranate seed oil 400 mg/day × 2	Paraffin 400 mg/day × 2	4 weeks	↓ TG, ↓ HDL-C, ↓ TG/HDL-C ratio, ↔ TNF-α	Asghari et al., 2012 [32]
Pre- and post-test	Hypertensive patients	M: 13, 39–68 years	Pomegranate juice 150 mL/day	-	1 day	↓ SBP, ↓ DBP, ↔ CRP, ↔ ICAM-1, ↔ VCAM-1, ↔ IL-6, ↔ E-selectin	Asgary et al., 2013 [33]
Pre- and post-test	Type 2 diabetic patients	F: 16, M: 15, 38–54 years	Concentrated pomegranate juice 50 g/day	-	4 weeks	↔ SBP, ↔ DBP, ↑ TC, ↑ HDL-C, ↔ TG, ↔ LDL-C, ↔ glycaemia, ↓ IL-6, ↔ TNF-α, ↔ CRP, ↓ adiponectin, ↑ TAC	Shishehbor et al., 2016 [34]
Double-blind, placebo-controlled, randomised crossover	Patients with metabolic syndrome	F: 16, M: 14, 42–62 years	Pomegranate juice 500 mL/day	Placebo 500 mL/day	1 week	↓ SBP, ↓ DBP, ↓ CRP, ↑ TG, ↑ VLDL-C	Moazzen & Alizadeh 2017 [35]
Double-blind, placebo-controlled, randomised	Haemodialysis patients	F: 46, M: 55, 55–81 years	Pomegranate juice 100 mL/day	Placebo 100 mL/day × 1	12 months	↓ IL-6, ↓ TNF-α, ↓ MPO, ↓ AOPP, ↓ oxidised fibrinogen, ↓ MDA	Shema-Didi et al., 2012 [36]
Pilot, open, randomised crossover	Haemodialysis patients	F: 13, M: 11, 47–75 years	Pomegranate juice 100 mL/day; Pomegranate extract POMx^TM^ 1050 mg/day (both containing 650 mg GAE)	-	4 weeks	↔ SBP, ↔ DBP, ↔ CRP, ↔ IL-6, ↔ F2-isoprostanes, ↔ isofurans, TG, ↔ TC, ↔ HDL-C, ↔ LDL-C	Rivara et al., 2015 [37]
Double-blind, placebo-controlled, randomised, parallel-arm	Haemodialysis patients	F: 10, M: 17, 49–59 years	Pomegranate extract POMx^TM^ 1 g/day (containing 600–755 mg GAE)	Placebo 1 pill/day	6 months	↓ SBP, ↓ DBP, ↔ CRP, ↔ IL-6, ↔ TC, ↔ HDL-C, ↔ LDL-C, ↔ TG, ↔ ORAC, ↔ AOPP, ↔ 8-OHdG, ↔ ox-LDL, ↔ arylesterase activity, ↔ lactonase activity, ↔ PON activity	Wu et al., 2015 [38]
Double-blind, placebo-controlled, randomised	RA patients	F & M: 55, 37–61 years	Pomegranate extract POMx^TM^ 250 mg/day × 2	Placebo (cellulose) 250 mg/day × 2	8 weeks	↔ CRP, ↔ MMP3, ↔ MDA, ↑ GPx, ↓ ESR, ↓ DAS28, ↓ HAQ, ↓ swollen joints, ↓ tender joints, ↓ pain intensity, ↓ morning stiffness	Ghavipour et al., 2017 [64]

↑: increase; ↔ : no change; ↓: decrease; 8-OHdG: 8-hydroxy-20-deoxyguanosine; AOPP: advanced oxidation protein products; CRP: C-reactive protein; DAS28: disease activity score; DBP: diastolic blood pressure; ESR: erythrocyte sedimentation rate; F: female; GAE: gallic acid equivalents; GPx: glutathione peroxidase; HAQ: health assessment questionnaire; HDL-C: high-density lipoprotein–cholesterol; ICAM-1: intracellular adhesion molecule-1; IL-6: interleukin 6; LDL-C: low-density lipoprotein–cholesterol; M: male; MDA: malondialdehyde; MMP3: matrix metalloproteinase-3; MPO: myeloperoxidase; ORAC: oxygen radical absorbance capacity; ox-LDL: oxidised low-density lipoprotein; PON: paraoxonases; SBP: systolic blood pressure; TAC: total antioxidant capacity; TC: total cholesterol; TG: triglycerides; TNF-α: tumour necrosis factor α; VCAM-1: vascular cell adhesion molecule-1; VLDL-C: very low-density lipoprotein–cholesterol.

**Table 3:** Ongoing clinical trials on pomegranate juice [72].

clintrials.gov Identifier	Study Focus	Study Design, Duration	Sponsor	Estimated Enrolment	Study Start Date	Estimated Completion Date
NCT02093130	Memory in older adults	Double-blind, placebo-controlled, parallel arm, randomised, 12 months	University of California (Los Angeles, CA, USA)	212	January 2014	December 2017
NCT02258776	Ageing and inflammation of the skin	Single-blind, placebo-controlled, parallel arm, randomised, 12 weeks	University of California (Los Angeles, CA, USA)	15	October 2015	January 2018
NCT03000101	Inflammation in IBD	Double-blind, placebo-controlled, parallel arm, randomised, 12 weeks	St. Orsola-Malpighi Hospital (Bologna, Italy)	36	December 2016	June 2018

**Table 4:** Overview of the anti-inflammatory effects of pomegranate or pomegranate-derived products in animal models of CID.

Disease Model	Animal Model	Tested Product(s), Vehicle, Duration	Disease Induction	Effects	Reference
Respiratory diseases	BalbC mice	Pomegranate peel aqueous extract (200 mg/kg b.w.) via intraperitoneal injection for 2 days	LPS-induced lung inflammation	↓ total cells in BAL, ↓ neutrophils in BAL	Bachoual et al., 2011 [17]
	BalbC mice	Encapsulated pomegranate leave extract (10 mg/mL) or non-encapsulated pomegranate leave extract (20 mg/kg b.w.) via nostril for 4 days	Ovalbumin-induced asthma	↓ leukocytes, neutrophils, and eosinophils in BAL, ↓ macrophages in BAL (non-encapsulated extract only), ↔ lymphocytes in BAL, ↓ IL-1β and IL-5 in BAL	de Oliveira et al., 2013 [18]
	C57BL/6J mice	Pomegranate juice (80 μmol/kg b.w.) via bottle for 1 week or 1 month or 3 months	Cigarette smoke-induced lung stress	↓ IL-1β and IL-6 expression in lung (1 week only), ↓ TNF-α expression in lung	Husari et al., 2016 [19]
IBD	Wistar rats	Punicic acid (400 mg/0.5 mL PBS) or pomegranate seed oil (0.5 mL) via oral administration for 10 days	TNBS-induced colitis	Punicic acid: ↓ Wallace and Ameho scores, ↓ MPO activity in colon, ↓ F2-isoprostane in colon; Pomegranate seed oil: ↓ Wallace and Ameho scores	Boussetta et al., 2009 [20]
	Swiss albino mice	Pomegranate flower hydro-alcoholic extract (100 or 200 mg/kg b.w.) or EA-rich fraction of pomegranate flower (100 or 200 mg/kg b.w.) via oral administration for 7 days	DSS-induced colitis	↓ macroscopic and histopathological changes in colon, ↓ colon MPO activity, ↓ histamine content in colon, ↓ MDA level in colon, ↓ superoxide anion production in colon	Singh et al., 2009 [27]
	Fischer rats	Pomegranate extract (250 mg/kg b.w.) or urolithin A (15 mg/kg b.w.) via chow for 10 days	DSS-induced colitis	↓ colon tissue damage (urolithin A only), ↑ FRAP (pomegranate extract only), ↓ MDA in colon (pomegranate extract only), ↓ COX-2 gene and protein expression in colon, ↓ iNOS expression in colon, ↓ PGE_2_ and NO levels in colon (pomegranate extract only), ↓ PTGES protein expression in colon	Larrosa et al., 2010 [22]
	Wistar rats	EA (10 and 20 mg/kg b.w.) via oral gavage for 48, 24 and 1 h prior to the induction of colitis and 24 h later	TNBS-induced colitis	↓ colon macroscopic damage, ↓ b.w. loss, ↓ colon weight/length, ↓ histological damage in colon, ↓ colon MPO activity, ↓ iNOS and COX-2 protein expression in colon, ↓ JNK and ERK phosphorylation in colon, ↓ NF-κB activation in colon	Rosillo et al., 2011 [25]
	Wistar rats	Pomegranate extract (250 or 500 mg/kg feed) or EA (10 mg/kg feed) or EA-enriched pomegranate extract (pomegranate extract 250 mg/kg feed + EA 10 mg/kg feed) via chow for 30 days prior to the induction of colitis and 14 days later	TNBS-induced colitis	↓ colon macroscopic damage, ↓ b.w. loss (all treatments, apart from extract 250 mg/kg), ↓ colon weight/length (EA and EA-enriched extract only), ↓ colon MPO activity, ↓ TNF-α level in colon, ↓ iNOS and COX-2 protein expression in colon, ↓ JNK and ERK phosphorylation in colon, ↓ NF-κB activation in colon, ↔ colon PPAR-γ protein expression	Rosillo et al., 2012 [24]
	C57BL/6 mice	EA (0.5% *w*/*w*, equivalent to 25 mg/mouse) via chow for 56 days	DSS-induced colitis	↓ disease symptoms, ↓ DAI, ↓ iNOS and COX-2 protein expression in colon, ↓ JNK and ERK phosphorylation in colon, ↓ NF-κB activation in colon, ↓ IL-6 gene expression in colon, ↓ STAT3 phosphorylation in colon	Marín et al., 2013 [23]
	Sprague-Dawley rats	Pomegranate beverage (containing 2504.74 mg/L GAE) *ad libitum* for 3 weeks prior to the induction of colitis and 7 weeks later	DSS-induced colitis	↓ colonocyte proliferative index, ↓ expression of hs-CRP, TNF-α, IL-1β, and IL-6 in intestinal mucosa, ↓ IL-1β and IL-6 levels in serum, ↑ IL-10 level in serum, ↓ p-p70-S6K/p70-S6K, ↓ p-rpS6/rpS6	Kim et al., 2016 [21]
	Sprague-Dawley rats	Pomegranate juice (400 mg/kg b.w.) or pomegranate powder (4 mg/kg b.w.) via oral administration for 18 days	DNBS-induced colitis	↔ histopathological scores, ↓ CMDI and DAI, ↓ MDA in colon (juice only), ↔ colon MPO activity, ↓ colon NO production, ↓ colon SOD activity, ↓ serum cortisol level, ↓ IL-1β, IL-18, TNF-α, and NF-κB expression in colon	Shah et al., 2016 [26]
Metabolic and cardiovascular disorders	Zucker rats	Concentrated pomegranate juice or pomegranate fruit extract (6.25 mL/L) via drinking water or pomegranate seed oil (1 mL/L) via chow for 5 weeks	Obese metabolic syndrome model	↔ TC, ↔ LDL-C, ↔ HDL-C, ↑ TG (seed oil only), ↔ daytime MAP, ↔ BPM, ↔ motor activity, ↓ arterial TSP-1 protein expression, ↑ eNOS protein expression (apart from oil), ↓ arterial TGF-β1 protein expression (except oil), ↓ nitrate and nitrite levels (apart from oil), ↔ insulin and glucose levels	de Nigris et al., 2007 [43]
	db/db mice	Pomegranate seed oil (1 g/100 g feed) via chow for 30 days	Diabetes and obesity model	↓ glycaemia, ↓ blood insulin, ↑ expression of genes PPAR-α, CD36, and FABP4 in adipose tissue, ↔ expression of genes PPAR-γ, ACAD, and SCD1 in adipose tissue, ↑ expression of genes PPAR-γ, CD36, FABP4, ACAD, and SCD1 in muscle, ↔ expression of genes PPAR-α in muscle, ↓ TNF-α expression and NF-κB activation in adipose tissue and liver	Hontecillas et al., 2009 [46]
	CD-1 mice	Pomegranate juice (12.5 mL/L juice diluted in water, equivalent to 0.35 mmol polyphenols) via drinking water for 4 months	Streptozotocin-induced diabetes	↑ hepatic PON-1 expression and activity, ↓ glycaemia, ↔ blood TC and TG levels	Betanzos-Cabrera et al., 2011 [41]
	Sprague-Dawley rats	Pomegranate juice (100 μL) via gastric gavage for 10 weeks	Streptozotocin-induced diabetes	↔ GSH in lung, ↑ SOD activity in lung, ↓ protein carbonyl content in lung, ↓ serum sialic acid, ↓ eNOS protein in lung	Çukurova et al., 2012 [42]
	SR-BI/apoE double knockout mice	Pomegranate extract (307.5 ml/L) via drinking water for 2 weeks	Coronary heart disease model	↑ TC, ↔ serum apoA and apoB, ↓ atherosclerosis, ↔ SAA and serum MCP-1, ↓ MCP-1 in plaques, ↓ lipid accumulation, macrophage infiltration, and MCP-1 levels in heart, ↓ myocardial fibrosis, cardiac enlargement, and ECG abnormalities	Al-Jarallah et al., 2013 [40]
	BalbC mice	Pomegranate peel extract (0.2% *w*/*v* diluted in water, equivalent to 6 mg per mouse) via drinking water for 4 weeks	High-fat diet-induced obesity and hypercholesterolaemia	↔ body weight gain, ↔ adiposity, ↔ glycaemia and insulin response, ↓ serum TC and LDL-C, ↔ serum HDL-C and TG, ↔ hepatic TC and TG, ↔ IL-1β, IL-6, and COX-2 expression in liver, ↔ IL-1β expression in colon, ↓ IL-6 and COX-2 expression in colon	Neyrinck et al., 2013 [47]
	Wistar rats	EA (0.8 g/kg feed) via chow for 8 weeks after the induction of metabolic syndrome	High-fat and high-carbohydrate diet-induced metabolic syndrome	↑ retroperitoneal, epididymal, omental, and total abdominal fat, ↔ whole-body fat mass, ↓ whole-body lean mass, ↓ glycaemia, ↓ plasma TG, TC, NEFA, uric acid, urea, and CRP, ↓ plasma ALT, AST, ALP, and LDH activity, ↔ plasma albumin and bilirubin, ↓ SBD, ↑ coronary endothelial-dependent relaxation, ↔ Nrf2 protein expression in heart, ↑ Nrf2 protein expression in liver, ↓ NF-κB expression in heart and liver, ↑ CPT1 expression in heart and liver	Panchal et al., 2013 [49]
	Wistar Albino Glaxo rats	Pomegranate extract (300 mg/kg b.w.) via chow for 8 weeks	High-fat diet-induced metabolic syndrome	↔ weight of epididymal adipose tissue, ↔ glycaemia, ↓ LDL-C, ↔ TC, HDL-C, TG, and FFA, ↔ SBP, ↓ serum corticosterone, ↔ adrenal corticosterone, ↓ serum IL-6 and TNF-α, ↓ TG in liver	Dushkin et al., 2014 [44]
	Sprague-Dawley rats	PUNI-enriched pomegranate extract (150 mg/kg b.w.) via oral gavage for 8 weeks	High-fat diet-induced NAFLD	↓ body weight gain, ↓ serum TG, HDL-C, and LDL-C, ↔ serum C, ↓ serum insulin, leptin, and adiponectin, ↓ HOMA-IR, ↓ serum ALT level, ↓ liver tissue weight, ↓ hepatic TG and TC, ↓ expression of SREBP-1c precursor protein, ↔ expression of SREBP-1c mature protein, expression of FA biosynthesis-related genes (↓ SREBP-1c, ↓ FAS, ↓ ACC1, ↓ SCD1), expression of TG biosynthesis-related genes (↓ ACLY, ↔ GPAM, ↑ DGAT-1 and-2), ↓ serum CRP level, IL-1β, IL-4, IL-6, and TNFα, ↓ serum IgA, IgG, and IgM, ↓ protein carbonyl content in liver tissue and liver mitochondria, ↓ lipid peroxidation in liver, ↑ hepatic total SOD activity, ↓ hepatic GSH and GSSG levels, ↑ GSH/GSSG ratio, ↓ Nrf2, HO-1, NQO-1, and UCP2 protein expression in liver, ↑ ATP content in liver, ↑ activities of mitochondrial complexes I, II, and IV in liver, ↑ expression of genes PGC-1-α and PPAR-α in liver, ↔ expression of PGC-1β gene in liver, ↑ PGC-1α protein expression in liver, ↑ expression of genes CPT1A, CPT1B, and ACAD in liver	Zou et al., 2014 [52]
	Pigs	Pomegranate extract Pomanox^®^ (625 mg equivalent to 200 mg punicalagins) via chow for 10 days	High-fat diet-induced coronary endothelial dysfunction	↑ coronary endothelial-dependent relaxation, ↑ Akt and eNOS phosphorylation in coronary artery, ↔ MCP-1 gene expression in coronary artery, ↓ MCP-1 protein content in coronary artery, ↓ coronary DNA oxidative damage, ↓ LDL-C oxidation	Vilahur et al., 2015 [51]
	Sprague-Dawley rats	Pomegranate juice concentrate (equivalent to 80 μmol polyphenols/mL) via drinking water for 5 weeks	Cigarette smoking-induced cardiac hypertrophy	↔ DBP and SBP, ↓ ROS in aortic tissue, ↓ heart to body weight ratio, ↓ fibrotic marker (ObR and Fn1) and kinin receptor (Bdkrb1 and Bdkrb2) expression in aorta, ↓ IL-1β expression in aorta, ↔ TNF-α expression in aorta	Al Hariri et al., 2016 [39]
	C57Bl/6 mice	Pomegranate peel (250 mg/kg b.w.) or Pomegranate flower extract (250 mg/kg b.w.) or Pomegranate seed oil (2 mL/kg b.w.) for 6 weeks	High-fat and high-sugar diet-induced obesity	↔ b.w. gain, ↓ glycaemia (28 days- seed oil treatment only), ↔ plasma insulin level, ↔ plasma TC, HDL-C, and TG, ↔ hepatic ALT and AST, ↔ hepatic TG, ↑ plasma IL-2 (peel extract only), ↓ plasma IL-6 (apart from flower extract), ↑ plasma IL-10 (flower extract only), ↓ plasma TNF-α (apart from peel extract), ↑ IFN-γ (seed oil only)	Harzallah et al., 2016 [45]
	Sprague-Dawley rats	Pomegranate juice (60 mL) via drinking water for 7 weeks	High-fat and high-sugar diet-induced NAFLD	↓ plasma ALT and AST, ↔ plasma GGT and ALP, ↓ glycaemia and insulin, ↓ plasma TG, ↔ plasma TC, HDL-C, and LDL-C, ↓ hepatic IL-1β, IL-6, TNF-α, and TGF-β1 expression, ↑ hepatic IL-10 expression, ↔ GSH level, TBARS level, GR activity, CAT activity, SOD activity in liver, ↑ hepatic GPx activity, ↓ hepatic steatosis and ballooning, ↓ lobular and portal inflammation in liver	Noori et al., 2017 [48]
	Sprague-Dawley rats	Pomegranate juice (1 mL) or pomegranate seed extract (100 mg/mL) via oral administration, by force-feeding for 21 days	Streptozotocin-nicotinamide induced type 2 diabetes	↔ b.w. gain, ↔ glycaemia and plasma insulin level, ↓ TC and TG (juice only), ↔ LDL-C and HDL-C (juice only), ↑ TC, LDL-C, and HDL-C (seed extract only), ↔ TG (seed extract only), ↓ plasma IL-6 and NF-κB levels, ↓ plasma TNF-α level (juice only), ↑ number and size of Islets of Langerhans (juice only)	Taheri Rouhi et al., 2017 [50]
Neurodegenerative diseases	APPswe/PS1dE9 mice	Pomegranate extract (6.25 mL/L) via drinking water for 3 months	Transgenic model overexpressing APP, developing amyloid plaques and progressive cognitive deficits	↑ behavioural performance, ↓ TNF-α in spleen and brain, ↓ NFATc1 activation in spleen and brain, ↑ p-NFATc2/NFATc2 ratio in brain, ↓ p-IκB/IκB ratio in brain, ↓ plaques in brain	Rojanathammanee et al., 2013 [59]
	Lewis rats	Pomegranate juice (juice diluted 1:40 in water, equivalent to ~0.6–0.7 mg polyphenols) via drinking water for 2 weeks	Rotenone-induced degeneration of neurones	↓ rearing behaviour, ↔ postural instability, ↔ catecholamine levels, ↓ dopamine fibres in striatum, ↓ nigral dopaminergic neurones, ↑ nitrotyrosine in substantia nigra, ↑ iNOS induction, ↑ NF-κB activation, ↑ caspase activation, ↔ IL-1β, TNF-α, and COX-2 protein expression	Tapias et al., 2014 [60]
	C57BL/6 mice	Pomegranate seed oil as emulsified nanodroplets (10 μL) via gavage for 10 days	MOG-induced experimental autoimmune encephalomyelitis	↓ demyelination and oxidation of brain lipids, ↓ MDA in brain	Binyamin et al., 2015 [54]
	APPsw/Tg2576 mice	Pomegranate fruit (4% *w*/*w*) via chow for 15 months	Transgenic model overexpressing APP, developing amyloid plaques and progressive cognitive deficits	↓ IL-2, IL-3, IL-4, IL-5, IL-9, IL-10, and eotaxin levels in serum, ↓ Aβ-1 40 and 42 levels in brain, ↑ ATP levels the cortex and hippocampus, ↓ IL-1β, IL-6, TNF-α levels in cortex and hippocampus	Essa et al., 2015 [56]
	APPsw/Tg2576 mice	Pomegranate fruit (4% *w*/*w*) via chow for 15 months	Transgenic model overexpressing APP, developing amyloid plaques and progressive cognitive deficits	↓ expression of genes IL-1β, IL-10, TNF-α, IGF-1, iNOS, and CCL2, ↑ BDNF gene expression, ↑ PSD-95, Munc18-1, SNAP25, and synaptophysin protein expression, ↑ p-CaMKIIα/CaMKIIα protein expression, ↑ p-CREB/CREB protein expression, ↑ BECN1 protein expression, ↑ LC3-I and LC3-II protein expression, ↑ Akt and mTOR protein expression, ↑ p70-S6K protein expression, ↔ APP and CTF-α protein expression, ↓ BACE-1, CTF-β, and sAPP-β protein expression, ↔ ADAM-10 and ADAM-17 protein expression	Braidy et al., 2016 [55]
	Wistar rats and mice	EA (10, 30, and 100 mg/kg b.w.) via intraperitoneal injection in a single administration	Scopolamine- and diazepam-induced cognitive impairments	↓ amnesia in EPM and PA tests in mice ([EA] ≥ 30 mg/kg), ↓ amnesia in EPM test in rats ([EA] ≥ 30 mg/kg)	Mansouri et al., 2016 [57]
	C57Bl/6 mice	Pomegranate peel extract as microparticles (800 mg/kg b.w.) via oral administration for 35 days	Amyloid-β peptide-induced neurodegeneration	↔ locomotor activity in an activity cage, ↔/↑ spatial memory in the Barnes maze, ↓ senile plaques, ↑ BDNF level in cortex and hippocampus, ↓ acetylcholinesterase activity in cortex and hippocampus, ↓ MDA in liver, ↔ SOD activity in hippocampus, cortex and serum, ↓ TNF-α in cortex, ↔ TNF-α in serum	Morzelle et al., 2016 [58]
	ICR mice	PUNI (1.5 mg/kg b.w.) via drinking water for 4 weeks	LPS-induced cognitive impairment	↓ Aβ and BACE-1 protein expression, ↓ GFAP and AIF-1 protein expression, ↓ IL-1β, IL-6, and TNF-α release, ↑ GSH/GSSG ratio, ↓ ROS level, ↓ MDA, ↓ IκB phosphorylation, ↓ p50 and p65 protein expression	Kim et al., 2017 [53]
RA	DBA/1 Lac J mice	POMx^TM^ extract (13.6 or 34 mg/kg b.w.) via oral gavage for 10 days	Collagen-induced arthritis with chicken CII (Chondrex)	↓ incidence and delay of arthritis, ↓ synovitis, ↓ pannus formation, ↓ joint degradation, ↓ IL-1β expression in ankle joints (13.6 mg/kg only), IL-6 expression in ankle joints, ↓ TNF-α expression in ankle joints (34 mg/kg only)	Shukla et al., 2008 [65]
Hepatocellular carcinoma	Sprague-Dawley rats	Pomegranate emulsion (1 or 10 g/kg b.w.) via oral gavage for 4 weeks prior to the DENA exposure and 18 weeks later	DENA-induced hepatocarcinogenesis	↓ cyclin D1 expression (10 g/kg only), ↑ Bax/Bcl-2 ratio (10 g/kg only), ↓ β-catenin expression (10 g/kg only), ↑ GSK-3 expression (10 g/kg only)	Bhatia et al., 2013 [67]
Prostatic hyperplasia	Sprague-Dawley rats	Pomegranate fruit extract (25, 50, and 100 mg/kg b.w.) via oral gavage for 4 weeks	Testosterone-induced prostatic hyperplasia	↓ prostate weight, ↓ PAP activity, ↑ GSH, ↔ total glutathione, ↑ SOD activity (100 mg/kg only), ↔ CAT activity, ↓ MDA, ↓ iNOS and COX-2 expression, ↔ AR, NF-κB, ER-α, and p-Akt expression	Ammar et al., 2015 [68]
Prostate cancer	Athymic nude mice	Pomegranate fruit extract (0.1% and 0.2% *w*/*v*) via oral administration for 28–51 days (until the implanted tumour reached to a volume of 1200 mm^3^)	Implantation with androgen-responsive CWR22Rn1 cells	↓ PSA secretion	Malik & Mukhtar 2006 [69]

↑: increase; ↔ : no change; ↓: decrease; ACAD: acyl coenzyme A dehydrogenase; ACC1: acetyl-CoA carboxylase 1; ACLY: ATP citrate lyase; ADAM: ADAM metallopeptidase; AIF-1: allograft inflammatory factor 1; Akt: protein kinase B; ALP: alkaline phosphatase; ALT: alanine transaminase; apoA: apolipoprotein A; apoB: apolipoprotein B; apoE: apolipoprotein E; APP: amyloid precursor protein; AR: androgen receptor; AST: aspartate aminotransferase; ATP: adenosine triphosphate; Aβ: amyloid β-peptides; b.w.: body weight; BACE-1: β-secretase 1; BAL: bronchoalveolar lavage; Bax: bcl-2-like protein 4; Bcl-2: B-cell lymphoma 2; Bdkrb: bradykinin receptor; BDNF: brain-derived neurotrophic factor; BECN1: beclin-1; BPM: beats per minute; CaMKIIα: calcium/calmodulin-dependent protein kinase type II α chain; CAT: catalase; CCL2: chemokine (C-C motif) ligand 2; CD36: cluster of differentiation 36; CMDI: colon mucosal damage index; COX-2: cyclooxygenase-2; CPT1: carnitine palmitoyl-transferase 1; CREB: cAMP response element-binding protein; CRP: C-reactive protein; CTF: C-terminal fragment of APP; CWR22Rn1: human prostate cancer cell line; DAI: disease activity index; DBP: diastolic blood pressure; DENA: diethyl-nitrosamine; DGAT: diglyceride acyltransferase; DNBS: 2,4-dinitro benzene sulfonic acid; DSS: dextran sulphate sodium; EA: ellagic acid; ECG: electrocardiogram; eNOS: endothelial nitric oxide synthase; EPM: elevated plus maze; ERK: extracellular signal-regulated kinase; ER-α: oestrogen receptor α; FA: fatty acids; FABP4: fatty acid binding protein 4; FAS: fatty acid synthase; FFA: free fatty acids; Fn1: fibronectin; FRAP: ferric reducing ability of plasma; GAE: gallic acid equivalents; GFAP: glial fibrillary acidic protein; GGT: γ-glutamyl-transferase; GPAM: glycerol-3-phosphate acyltransferase; GPx: glutathione peroxidase; GR: glutathione reductase; GSH: reduced glutathione; GSSG: oxidised glutathione; h: hour(s); HDL-C: high-density lipoprotein-cholesterol; HO-1: heme oxygenase 1; HOMA-IR: homeostatic model assessment–insulin resistance; hs-CRP: high-sensitivity C-reactive protein; ICR: imprinting control region; IgA: immunoglobulin A; IGF-1: insulin-like growth factor 1; IgG: immunoglobulin G; IgM: immunoglobulin M; IL-1β: interleukin 1β; IL-2: interleukin 2; IL-3: interleukin 3; IL-4: interleukin 4; IL-5: interleukin 5; IL-6: interleukin 6; IL-9: interleukin 9; IL-10: interleukin 10; IL-18: interleukin 18; iNOS: inducible nitric oxide synthase; IκB: inhibitor of NF-κB; JNK: c-Jun N-terminal kinase; LC3: microtubule-associated protein 1α/1β-light chain 3; LC3-I: cytosolic form of LC3; LC3-II: LC3-phosphatidylethanolamine conjugated; LDH: lactate dehydrogenase; LDL-C: low-density lipoprotein-cholesterol; LPS: lipopolysaccharide; m: month(s); MAP: mean arterial pressure; MCP-1: monocyte chemoattractant protein-1; MDA: malondialdehyde; MOG: myelin oligodendrocyte glycoprotein; MPO: myeloperoxidase; mTOR: mechanistic target of rapamycin; Munc-18: syntaxin binding protein 1; NAFLD: non-alcoholic fatty liver disease; NEFA: non-esterified fatty acids; NFATc: nuclear factor of activated T-cells, cytoplasmic; NF-κB: nuclear factor κ light-chain-enhancer of activated B cells; NO: nitric oxide; NQO-1: NAD(P)H quinone dehydrogenase 1; Nrf2: nuclear factor (erythroid-derived-2)-like 2; ObR: leptin receptor; p-: phosphorylated; p50: p50 protein; p65: p 65 protein; p70-S6K: ribosomal protein S6 kinase β-1; PA: passive avoidance; PAP: prostatic acid phosphatase; PBS: phosphate-buffered saline; PGC-1-: peroxisomal proliferator-activated receptor-γ coactivator-1; PGE_2_: prostaglandin E2; PON-1: paraoxonase 1; PPAR-α: peroxisome proliferator-activated receptor α; PPAR-γ: peroxisome proliferator-activated receptor γ; PSA: prostate-specific antigen; PSD-95: postsynaptic density protein 95; PTGES: prostaglandin E synthase; PUNI: punicalagin; ROS: reactive oxygen species; rpS6: ribosomal protein S6; SAA: serum amyloid A; sAPP-β: soluble APP-β; SBP: systolic blood pressure; SCD1: stearoyl-coenzyme A desaturase 1; SNAP25: synaptosomal-associated protein 25; SOD: superoxide dismutase; SR-BI: scavenger receptor class B type I; SREBP-1c: sterol regulatory element-binding protein 1c; STAT3: signal transducer and activator of transcription 3; TC: total cholesterol; TG: triglycerides; TGF-β1: transforming growth factor β1; TNBS: 2,4,6-trinitrobenzenesulfonic acid; TNF-α: tumour necrosis factor α; TSP-1: thrombospondin 1; UCP2: mitochondrial uncoupling protein 2; *w*/*v*: weight/volume; *w*/*w*: weight/weight.

**Table 5:** Summary of the anti-inflammatory effects of pomegranate extracts or pomegranate-derived bioactive compounds assayed in cell culture studies.

Cell Model	Primary Cell/Cell Line	Tested Compound(s), Dose, Duration	Pro-Inflammatory Treatment	Biological Effects	Reference
Intestinal cells	CCD18-Co	Uro-A (40 μM) + Uro-B (5 μM) + EA (1 μM) for 12–48 h in concomitant exposure with pro-inflammatory stimulus	IL-1β (1 ng/mL) or TNF-α (50 ng/mL)	↓ IL-8 release, ↓ PGE_2_ release (only upon IL-1β stimulus), ↓ PAI-1 release, ↔ ICAM-1 and VCAM-1 release, ↔ MCP-1, ↓ cell migration and adhesion	Giménez-Bastida et al., 2012 [28]
	Caco-2	Pomegranate husk extract (containing 8.1 μM PUNI and 7.9 μM EA) or PUNI (50 μM) for 1 h as pre-treatment and 24 h in concomitant exposure with pro-inflammatory stimulus	basolateral side: IL-1β (25 μg/L) + TNF-α (50 μg/L) + IFN-γ (50 μg/L); apical side: LPS (1 mg/L)	↓ IL-6 and MCP-1transcription, ↔ IL-8 transcription, ↓ IL-6, IL-8, and MCP-1 secretion	Hollebeeck et al., 2012 [29]
Immune cells	KU812	POMx^TM^ extract (20, 40, and 100 μg/mL) for 2 h prior to pro-inflammatory stimulus	PMA (40 nM) + A23187 (1 μM)	↓ IL-6 and IL-8 transcription, ↓ IL-6 and IL-8 secretion, ↓ JNK and ERK phosphorylation, ↓ NF-κB activation	Rasheed et al., 2009 [31]
	Primary HGE	EA (12.5, 25, 50, and 100 μM) for 18 h	-	↓ IL-8 transcription ([EA] ≥ 25 μM), ↑ BD2 transcription ([EA] ≥ 25 μM), ↑ SLPI transcription, ↓ CCL20 transcription ([EA] ≥ 50 μM), ↓ CXCL5 transcription ([EA] ≥ 50 μM), ↔ IL-1β secretion, ↑ IL-2 secretion ([EA] = 12.5 μM), ↓ IL-2 secretion ([EA] = 50 μM), ↔ IL-4, IL-6, and TNF-α secretion, ↓ IL-8 secretion ([EA] = 50 μM), ↔ MCP-1 secretion, ↑ CCL5 secretion ([EA] ≥ 12.5 μM), ↑ BD2 secretion ([EA] = 100 μM), ↔ SLPI secretion	Promsong et al., 2015 [30]
Neuronal cells	PC12	Pulp aqueous extract (6.25–800 μg/mL), pulp hydro-alcoholic extract (6.25, 12.5, 25, 50, 100, 200, 400, and 800 μg/mL), PJ extract (6.25, 12.5, 25, 50, 100, 200, 400, and 800 μg/mL) for 2 h prior glucose deprivation	Serum glucose deprivation	↓ DNA damage ([PJ] ≥ 400 μg/mL)	Forouzanfar et al., 2013 [61]
	BV-2	Pomegranate seed oil (25 μg/mL) for 24 h	LPS (1 mg/mL)	↓ NO production, ↓ TNF-α release, ↓ iNOS induction, ↓ caspase 3 activation	Račková et al., 2014 [62]
	SK-N-SH	PJ extract (25, 50, 100, and 200 μg/mL) for 24 h	IL-1β (10 U/mL)	↓ PGE_2_ release, ↓ COX-2 protein expression, ↓ BACE-1 ([PJ] ≥ 50 μg/mL), ↓ amyloid-β ([PJ] ≥ 100 μg/mL), ↓ IκBα phosphorylation ([PJ] ≥ 50 μg/mL)	Velagapudi et al., 2016 [63]
	Primary astrocytes and BV-2	PUNI (10, 20, and 50 μM) for 1 h	LPS (1 mg/mL)	↓ iNOS and COX-2 protein expression, ↓ APP and BACE-1 protein expression, ↓ IκBα phosphorylation	Kim et al., 2017 [53]
Rheumatoid arthritis cells	MH7A	Delphinidin (10 and 30 μM) for 24 h or 2 h (for ELISA)	TNF-α (20 ng/mL)	↓ IL-1β and IL-6 expression, ↓ COX-2 expression, ↓ p65 acetylation, ↓ NF-κB DNA binding activity	Seong et al., 2011 [66]
Cancer cells	DU145 and PC3	PJ (1% or 5%) for 18 h	-	↓ IL-6 and IL-12 secretion, ↓ IL-1β secretion (DU145 only), ↓ CCL5 secretion (PC3 only)	Wang et al., 2011 [70]

↑: increase; ↔ : no change; ↓: decrease; APP: amyloid precursor protein; BACE-1: β-secretase 1; BD2: β-defensin-2; BV-2: murine microglial cell line; Caco-2: human colorectal adenocarcinoma cell line; CCD18-Co: human colon cell line; CCL5: chemokine (C-C motif) ligand 5; CCL20 chemokine (C-C motif) ligand 20; COX-2: cyclooxygenase-2; CXCL5: chemokine (C-X-C motif) ligand 5; DU145: human prostate carcinoma cell line; EA: ellagic acid; ELISA: enzyme-linked immunosorbent assay; ERK: extracellular signal–regulated kinase; h: hour(s); ICAM-1: intracellular adhesion molecule-1; IFN-γ: interferon γ; IL-1β: interleukin 1β; IL-2: interleukin 2; IL-4: interleukin 4; IL-6: interleukin 6; IL-8: interleukin 8; iNOS: inducible nitric oxide synthase; IκB: inhibitor of NF-κB; JNK: c-Jun N-terminal kinase; KU812: human basophilic leukaemia cell line; LPS: lipopolysaccharide; MCP-1: monocyte chemoattractant protein-1; MH7A: human rheumatoid arthritis synovial cell line; NF-κB: nuclear factor κ light-chain-enhancer of activated B cells; NO: nitric oxide; P65: transcription factor p65; PAI-1: plasminogen activator inhibitor-1; PC3: human prostate cancer cell line; PC12: rat adrenal gland cell line; PGE_2_: prostaglandin E_2_; PJ: pomegranate juice; PMA: phorbol 12-myristate 13-acetate; PUNI: punicalagin; SK-N-SH: human neuroblastoma cell line; SLPI: secretory leukocyte protease inhibitor; TNF-α: tumour necrosis factor α; Uro-A: urolithin A; Uro-B: urolithin B; VCAM-1: vascular cell adhesion molecule-1.
